# How Times Have Changed! A Cornucopia of Antigens for Membranous Nephropathy

**DOI:** 10.3389/fimmu.2021.800242

**Published:** 2021-11-26

**Authors:** Tiffany N. Caza, Laith F. Al-Rabadi, Laurence H. Beck

**Affiliations:** ^1^ Arkana Laboratories, Little Rock, AR, United States; ^2^ Department of Internal Medicine (Nephrology & Hypertension), University of Utah, Salt Lake City, UT, United States; ^3^ Department of Medicine (Nephrology), Boston University School of Medicine and Boston Medical Center, Boston, MA, United States

**Keywords:** membranous nephropathy, membranous lupus nephritis, antigen, epitope spreading, serologic testing, autoimmune profiling, mass spectrometry

## Abstract

The identification of the major target antigen phospholipase A2 receptor (PLA2R) in the majority of primary (idiopathic) cases of membranous nephropathy (MN) has been followed by the rapid identification of numerous minor antigens that appear to define phenotypically distinct forms of disease. This article serves to review all the known antigens that have been shown to localize to subepithelial deposits in MN, as well as the distinctive characteristics associated with each subtype of MN. We will also shed light on the novel proteomic approaches that have allowed identification of the most recent antigens. The paradigm of an antigen normally expressed on the podocyte cell surface leading to *in-situ* immune complex formation, complement activation, and subsequent podocyte injury will be discussed and challenged in light of the current repertoire of multiple MN antigens. Since disease phenotypes associated with each individual target antigens can often blur the distinction between primary and secondary disease, we encourage the use of antigen-based classification of membranous nephropathy.

## Introduction

Membranous nephropathy (MN) is an autoimmune kidney disease that is the second leading cause of nephrotic syndrome. The pathological hallmark of MN is the deposition of immunoglobulin G (IgG) and variable amounts of complement proteins within the subepithelial space. Immune complexes can form *in situ* due to circulating antibodies targeting an intrinsic or planted antigen within the glomeruli or from deposition of immune complexes that form in the circulation and then become trapped in the subepithelial space (1, 2). The specific antigens within these immune complexes have been progressively identified through technological advancements.

Analogous to Moore’s Law which predicted an exponential increase in computing power as technology allowed more transistors per chip, MN research has recently been marked by an accelerated pace in the discovery and identification of antigens, more so than at any time since the first delineation of MN as a distinct clinicopathologic entity in 1957 (3). The research journey to uncover the underlying mysteries of MN is best described by Couser in the title of a 2005 review article entitled “Membranous Nephropathy: A Long Road But Well Traveled (4)”. It is worthwhile to review this journey within the context of a wave of recent advances in MN research, which has identified new antigens and highlighted new methodologies for such discoveries.

Early studies questioned whether the source of the subepithelial immune deposits in MN was from immune complexes that formed in the circulation and then deposited in the glomeruli (which was the prevailing view) or if, instead, they formed *in situ* (5). Similarly, whether these antigens were normally expressed in the glomerulus, and by which cell type, or were extrinsic proteins that became implanted in the glomerular basement membrane (GBM) (6, 7) or beneath the podocyte was not clear. An animal model known as Heymann nephritis provided an excellent investigatory platform and early efforts focused on replicating features of the disease process in this experimental rat model to better understand human MN pathogenesis (8). This particular model involved immunizing rats with a fraction of proximal tubular brush border, which resulted in a histopathological pattern of MN nearly identical to what was seen in human glomeruli. Two groups independently disproved the reigning hypothesis that circulating immune complexes were responsible for the glomerular deposits and showed evidence for *in situ* formation of the deposits, with circulating antibodies targeting an intrinsic antigen of the glomerular filtration barrier (9, 10). While these data support *in situ* immune complex formation/planted antigens, the existence of circulating complexes has not been disproved. It has been proposed that circulating immune complexes can occur in the setting of membranous nephropathy related to autoimmune disease, such as membranous lupus nephritis, but has not been proven in humans or animal models. The brush border component that triggered immunogenicity in the Heymann nephritis model was ultimately identified as megalin, also known as low density lipoprotein-related protein 2 (LRP2) (11). Despite the similarities between Heymann nephritis and human MN, megalin could not be shown to represent the target antigen in human disease (12). Of note, LRP2 was later identified to be the main antigen in the tubular basement membrane immune deposits in anti-brush border antibody disease (anti-LRP2 nephropathy), which is associated with MN features (13).

Other animal models of MN had been developed, including those utilizing exogenous cationic bovine serum albumin (BSA) as an antigen that traversed the GBM and become planted in a subepithelial space. Mice injected with cationic BSA developed features of MN with proteinuria developing within two weeks (14). This modified protein was later identified to represent an actual antigen in a minority of human cases of childhood MN associated with antibodies to cationic BSA (6). Other murine models implicated antibodies to dipeptidyl peptidase IV (15), aminopeptidase A (APA) (16), α3NC1 (17), and annexin 3 (18), but corresponding antigens in humans have not been identified to date.

The first evidence of an intrinsic glomerular antigen in human MN came through experiments of nature. Ronco and colleagues documented rare cases of antenatal MN occurring in newborns of mothers deficient in neutral endopeptidase (NEP), a known glomerular protein (1). These mothers developed alloantibodies to NEP which led to subepithelial deposits in the fetal kidney (which expressed NEP due to paternal inheritance); the alloantibodies disappeared several months after birth and were associated with clinical resolution of disease in the infant (1). This finding confirmed that circulating antibodies directed against a podocyte antigen could lead to MN in humans. Yet the target antigen in adult disease, representing the vast majority of MN, remained unknown.

It was not until 2009 that a target antigen was identified in adult MN. Using patient serum to probe immunoblots of human glomerular proteins, Beck and Salant were able to find evidence of a common 185 kDa protein identified by the serum from a proportion of idiopathic MN cases. Mass spectrometric analysis of the gel band revealed this protein as the M-type (muscle derived) phospholipase A2 receptor (PLA2R) (19). PLA2R was thus the first identified target antigen in adult MN, and is now known to be the most commonly-targeted antigen, associated with 70-80% of MN cases (19). A second minor antigen, thrombospondin type 1 domain-containing 7A (THSD7A) was identified several years later using similar methodology (20).

Despite the identification of THSD7A and PLA2R, the specific target antigens in approximately 30% of primary MN cases remained unknown. There was a need to further characterize the antibody repertoire in MN for patients who were PLA2R and THSD7A-negative. However, for rare antigens, it was becoming increasingly difficult to identify common pattern of reactivity by Western blotting, confounded by a proportion of patients who are in immunologic remission at the time of serum sampling and therefore would not have these rare circulating autoantibodies. Therefore, new approaches were necessary that instead focused on the kidney biopsy as a tissue source to identify the antigen, under the assumption that the targeted antigen would be enriched in the subepithelial immune complexes relative to normal glomerular proteins (21, 22).

## Technologies for Antigen Identification

**Figure 1:**
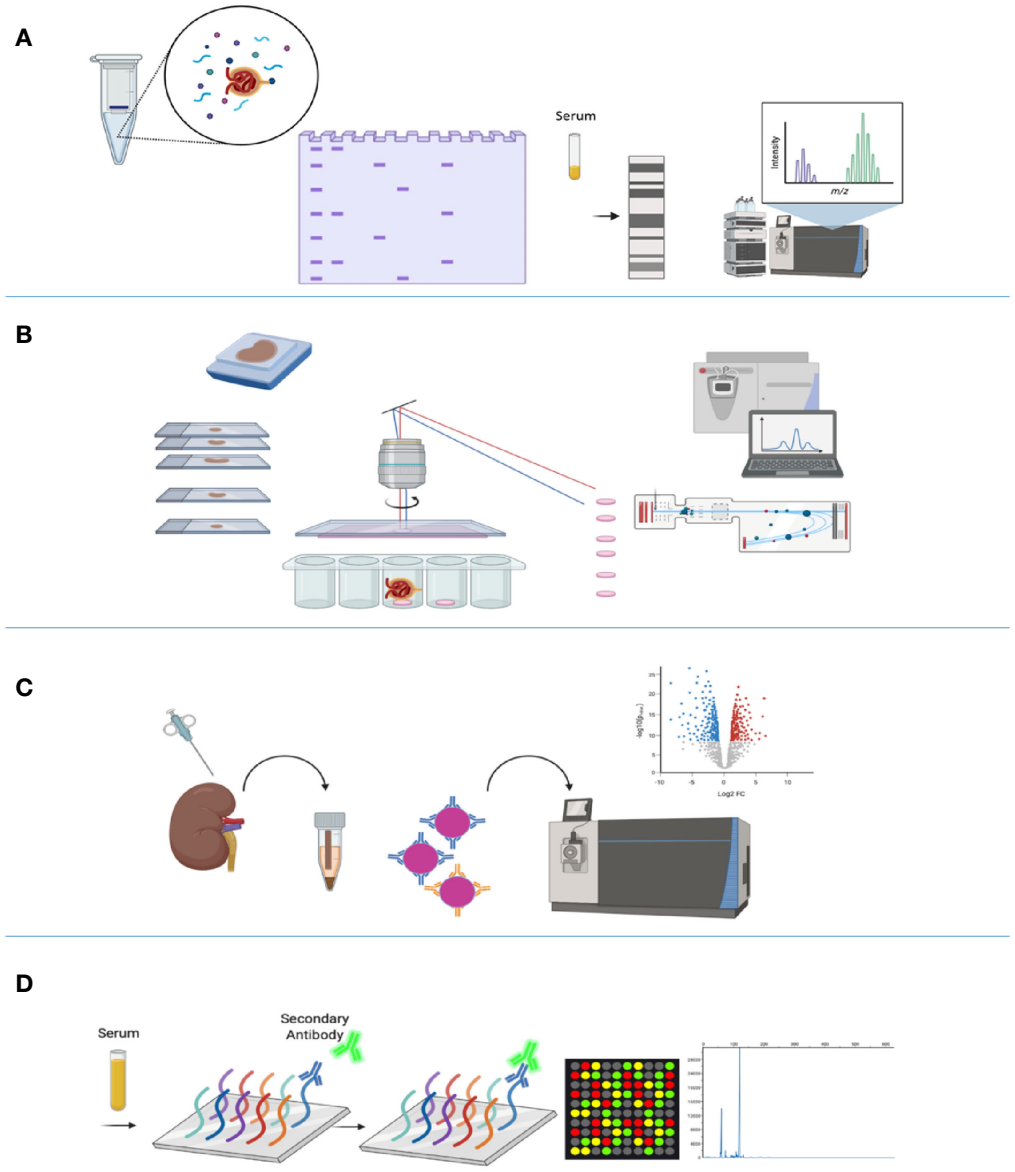
Technologies used in the identification of membranous nephropathy antigens. **(A)** Western blotting evaluates seroreactivity against human glomerular extract to identify podocyte antigens by mass spectrometry. **(B)** Laser capture microdissection (LCMD) from kidney biopsy specimens enriches for glomerular proteins, which is followed by mass spectrometry for protein identification. **(C)** Protein G immunoprecipitation (tissue IP) from frozen tissue enriches for immune complexes in kidney biopsy tissue for protein identification by mass spectrometry. **(D)** Autoimmune profiling evaluates seroreactivity against an array of peptides to identify potential autoantigens. Created with BioRender.com.

Western blotting for the detection of glomerular antigens remains an effective method by which to identify additional candidate antigens and to further validate tissue-based discovery. Moreover, analysis of bands differentially recognized by serial serum samples under different clinical states (nephrosis *vs*. remission *vs*. relapse) (19, 22, 23), followed by immunoprecipitation and analysis by MS remains a viable technique for antigen identification (**Figure 1A**).

Recent advances in proteomic technologies have enabled large-scale profiling of proteins in tissue and sera from patients, with the potential to better diagnose and classify autoimmune diseases such as MN (24). The powerful method of laser capture microdissection (LCM) of glomeruli from formalin-fixed paraffin-embedded biopsy tissue, followed by mass spectrometric (MS) proteomic analysis, capitalizes on enrichment of glomerular proteins or antigens in certain disease states (25, 26) (**Figure 1B**). Multiple kidney diseases have been better defined through LCM-MS, including membranoproliferative glomerulonephritis (GN), amyloidosis, cryoglobulinemic GN, fibrillary GN, and membranous-like glomerulopathy with masked IgG kappa deposits (MGMID) (25–29).

In the field of MN, the use of sequential methods of LCM followed by MS has allowed the discovery of multiple new autoantigens including the exostosin 1/2 complex (EXT1/2) (21), neural epidermal growth factor-like 1 (NELL1) (22), semaphorin 3B (SEMA3B) (30), and protocadherin 7 (PCDH7) (31). It was also utilized as an ancillary technique in confirmation of the new autoantigens serine protease HTRA1 (HTRA1) (23), neural cell adhesion molecule 1 (NCAM1) (32), and type III transforming growth factor-beta receptor (TGFBR3) (33). Additionally, the LCM-MS methodology confirmed the appropriate target antigen in cases of PLA2R- and THSD7A-associated MN cases, validating its utility for all known subtypes of MN.

This technique was subsequently followed by protein G immunoprecipitation (IP) from biopsy tissue, specifically focusing on immune complexes eluted from kidney tissue, followed by MS for identification of antigenic targets (**Figure 1C**). This tissue IP was the main method of identification of NCAM1 and TGFBR3, and additionally supported the discovery of HTRA1 (23). Functional validation of targeted antigens was performed by co-localization of each target antigen with IgG within the glomerular immune deposits.

Autoimmune profiling, based on serum reactivity with whole proteome arrays, has emerged as a promising methodology to complement the previously mentioned modalities (**Figure 1D**). Autoimmune profiling, a new technology based on peptide or protein fragment microarrays for the analysis of the antibody repertoire in various autoimmune conditions, has only been implemented in a few studies (34–36). This technique has been applied to MN as an ancillary technique for the discovery of autoantibodies against a novel MN antigen serine protease HTRA1 (23), demonstrating proof-of-concept for its use in this disease.

An overview of these methods is shown in **Figure 1**.

## Autoantigens in Primary MN

Traditionally, MN has been classified as a primary (previously ‘idiopathic’) or secondary disease. The presence of humoral autoreactivity against a known antigen (*e.g.*, PLA2R or THSD7A) has been considered to represent primary MN (37) while the association of MN with various concurrent conditions such as malignancy, systemic autoimmune diseases, infections, or medications has been considered secondary, especially in the absence of staining for discrete MN antigens (38). While many of these secondary causes have consistently been associated with MN, in some cases it is difficult to exclude the coincidental occurrence of primary MN and another condition (39).

**Figure 2:**
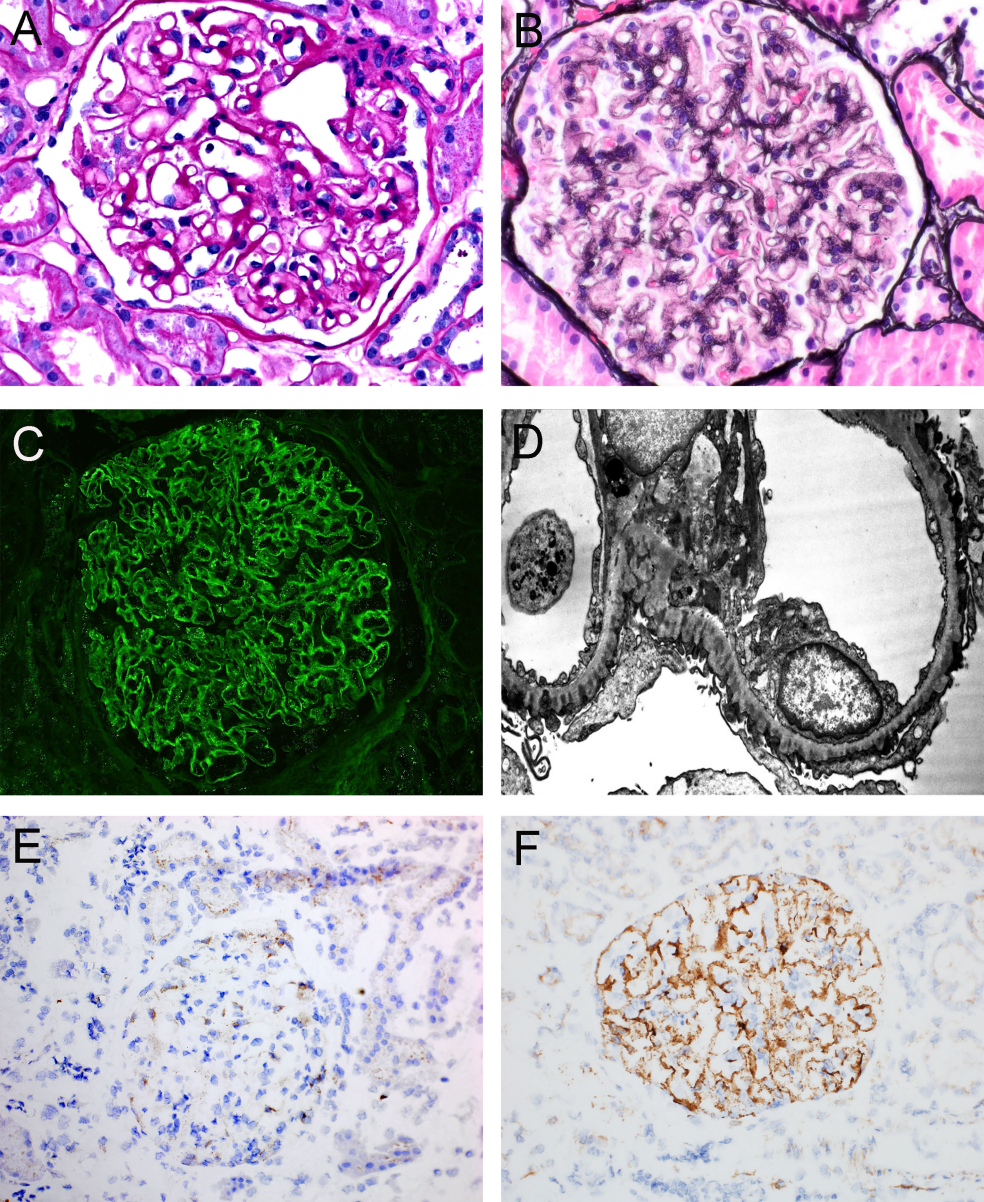
Representative histopathologic features of membranous nephropathy. **(A)** Glomerulus with prominent glomerular capillary loops, PAS, 400x. **(B)** Glomerulus with capillary loop holes/lucencies, Jones Methenamine Silver, 400x. **(C)** Global granular capillary loop staining, IgG immunofluorescence, 400x. **(D)** Electron photomicrograph of subepithelial and intramembranous electron-dense deposits. **(E)** Immunohistochemistry of a case of PLA2R-negative MN, demonstrating staining within podocyte cell bodies consistent with inherent low-level PLA2R expression, 200x. **(F)** Immunohistochemistry of a case of PLA2R-positive MN, demonstrating global granular capillary loop staining (positive result), 200x. Created with BioRender.com.

Features on kidney biopsy can often point to primary or secondary disease. The histopathology of primary MN, typically evident with PLA2R- and THSD7A-associated disease, demonstrates diffuse and global granular capillary loop staining for IgG with C3, but not C1q or other immunoglobulin heavy chains, within glomeruli. Ultrastructurally, subepithelial and intramembranous electron-dense deposits are present, with the absence of mesangial or subendothelial deposits (40) (**Figure 2**). IgG subclass staining, when performed, demonstrates a predominance of IgG4 in primary MN (41–44).

While the field may be moving away from a categorization of primary *vs*. secondary MN and more towards an antigen-based classification system (45, 46), we will continue to use the terms ‘primary’ and ‘secondary’ in this review when applicable. We will describe both podocyte antigens, including PLA2R, THSD7A, HTRA1, and SEMA3B as well as non-podocyte antigens including NELL1 and PCDH7, in what we still call ‘primary’ membranous nephropathy. This will be followed by antigens commonly encountered in membranous lupus nephritis, including the podocyte-expressed antigens NCAM1, TGFBR3, and CNTN1 as well as the more ubiquitously-expressed proteins EXT1 and EXT2.

## Primary MN Antigens Expressed Within Podocytes

### Phospholipase A2 Receptor

PLA2R is a transmembrane glycoprotein and member of the small mannose-receptor family that is expressed in podocytes and in other tissues (47). PLA2R is comprised of multiple domains, and the conformation of many of these domains is maintained by regular patterns of disulfide bonding (48). The extracellular region comprises an N-terminal cysteine-rich (CysR or ricin B) domain, a single fibronectin type-2 (FnII) domain, and eight C-type lectin-like domains (CTLD). Motifs present in the short cytoplasmic domain enable constitutive endocytic recycling in clathrin-coated pits. PLA2R undergoes pH-dependent conformational changes that are necessary for ligand binding and subsequent release of the ligand in the more acidic pH of endosomes and lysosomes (48, 49). Patients often have a genetic predisposition with risk alleles in both MHC class II genes and in the *PLA2R1* gene itself (50). Patients with PLA2R-positive MN had a mean age of 58 years and had male predominance (19). Kidney biopsies of patients with PLA2R-positive MN showed diffuse and global granular capillary loop staining for IgG, C3, and light chains and did not show significant expression for other immune reactants.

Autoantibodies are reactive against the protein in its native and non-reduced conformation, with seroreactivity lost under reducing conditions (19). PLA2R antibodies, predominantly of the IgG4 subclass (19), correlate with disease activity and remission and are useful to evaluate treatment decisions and evaluate prognosis (51–53). Epitope spreading from the N-terminal cysteine-rich (CysR) domain to C-type lectin-like domains (CTLD1-8) has been identified in several studies and is associated with a poor prognosis (54, 55). Dependence of autoantibody binding to PLA2R and other proteins under native conditions with pH and/or disulfide bond dependence has been based on immunoblotting experiments under reducing and non-reducing conditions. For PLA2R, this was supported by further epitope identification requiring non-reducing conditions, however work towards epitope identification has not been extensively studied for most autoantigens. However, this was investigated with similar requirements for native conditions found for the second identified primary MN autoantigen, THSD7A.

### Thrombospondin Type 1 Domain-Containing 7A

THSD7A was the second autoantigen discovered in primary MN, identified after recognition that MN sera uncommonly showed reactivity by immunoblotting with a 250 kDa antigen present in human glomerular protein extracts that was subsequently identified as THSD7A (20). THSD7A is also a large glycoprotein expressed by the podocyte, and stimulates an IgG4-predominant autoantibody response in 3%–5% of patients with primary MN (20). Similar to PLA2R, the epitopes within THSD7A are sensitive to reducing agents (20). Autoantibodies against THSD7A recognize multiple protein domains within the protein, with the N-terminal end of the protein being the predominant region (56).

Patients with THSD7A-associated MN had a mean age of 62 years and had a slight male predominance (57). Similar to PLA2R-associated MN, there was diffuse and global granular capillary loop staining for IgG, C3, and light chains without significant staining for other immune reactants. THSD7A-positive MN is not strongly associated with underlying autoimmune diseases (such as systemic lupus erythematosus) but is seen in some patients with malignancy, where there is corresponding increased expression in tumor tissue (58, 59). Anti-THSD7A antibodies correlate with disease activity (60, 61) and serologic testing is routinely used in clinical practice (56). Further details regarding PLA2R and THSD7A MN have been discussed in a prior review (62).

### Serine Protease HTRA1

HTRA1 was identified by an inter-institutional collaborative group including these three authors, utilizing four independent but adjunctive methodologies (23). Western blotting of human glomerular proteins with serum from the index patient identified a 50 kDa candidate antigen and mass spectrometry of immunoprecipitates from serum at the time of nephrosis but not remission revealed the identity of this protein as the serine protease HTRA1. This finding was independently confirmed by tissue-based studies with LCM-MS and tissue IP-MS, as well as by autoimmune profiling of nephrotic *versus* remission sera from the index patient. HTRA1 is a secreted trypsin-like serine protease that is involved in the homeostasis of the extracellular matrix.

HTRA1-associated MN comprises a total of 4.2% of PLA2R-, THSD7A-, NELL1-, and EXT2-negative cases, with the caveat that it represents a much smaller proportion of the entire primary MN spectrum. It also appears to be a disease of elderly, with a mean age of 67 years in this US-based cohort. Similar to PLA2R- and THSD7A-associated MN, there is diffuse and global capillary loop IgG staining on biopsy, with expression of IgG and C3, but not other immune reactants. IgG4 was the dominant IgG subclass. HTRA1 was specifically detected within immune deposits of HTRA1-associated MN but not in other types of MN. HTRA1-associated MN is favored to be mostly primary, based on the lack of a clinical history of autoimmune or infectious disease and negative staining in MLN biopsies. Circulating anti-HTRA1 antibodies recognized native and recombinant protein under both reducing and non-reducing conditions, suggesting a linear epitope and lack of disulfide bond dependence. Longitudinal measurements of anti-HTRA1 antibodies by immunoblotting showed an apparent correlation with disease course, suggesting serial monitoring might assist in therapeutic decision making.

### Semaphorin 3B

SEMA3B was also identified in primary MN by LCM-MS and is enriched within cohorts of pediatric MN patients, some of which were less than 2 years of age (30). SEMA3B is part of the semaphorin family of proteins, which have high expression in the nervous system and, similar to NELL1, is highly expressed during development (63). It is expressed in podocytes, as demonstrated from protein expression in the Human Protein Atlas (64, 65) and single cell RNA sequencing analysis data (66). A related protein, semaphorin 3A, has been associated with podocyte foot process effacement and proteinuria (67), although the function of the related SEMA3B in the kidney is unknown.

In the discovery cohort by Sethi et al., eight of 11 cases of SEMA3B-associated MN were in pediatric patients (30), making up approximately 10% of MN biopsies in this population, while this form of MN was found to occur rarely in adult MN (<1%). Nearly all patients in this report had achieved partial or complete clinical remission at last follow-up (30). Unlike PLA2R-, THSD7A-, and NELL1-associated MN, IgA and/or C1q staining of the glomerular deposits has been demonstrated in some patients. Approximately one-third of the pediatric cases also had tubular basement membrane (TBM) deposits. IgG1 is the predominant IgG subclass (30). A possibility of familial predilection was discussed in this report, based on two cases.

The presence of SEMA3B autoantibodies in the circulation was demonstrated by serum reactivity against the recombinant protein. Interestingly, and unlike the case for autoantibodies against PLA2R and THSD7A, the serum from these cases reacted only with the reduced form of SEMA3B and not the non-reduced protein (30). This raises the question of how the autoantibody would react with the protein in its native configuration and whether another mechanism (or genetic variant) would be required to expose this cryptic epitope.

## Primary MN Antigens Not Expressed Within Podocytes

### Neural Epidermal Growth Factor-Like 1

NELL1 was identified as the first antigen in non-lupus related MN initially using LCM-MS (22) and later by tissue IP (68). NELL1 is a non-membrane bound glycoprotein that is expressed during development and primarily expressed within the nervous system (69). Unlike the case for the target antigens in PLA2R and THSD7A-positive MN, NELL1 is not routinely expressed by podocytes under normal conditions. NELL1 associated MN comprises 3.8-16% of all PLA2R-negative MN cases in the United States and may be more frequent among Chinese patients, making up 35% of PLA2R-negative MN cases in these cohorts (22, 68, 70).

NELL1-associated MN has unique histopathologic features, including a segmental or incomplete global pattern of immune complex deposition (71) and IgG1 subclass predominance (22, 68, 71). The presence of NELL1 autoantibodies in the circulation was demonstrated by seroreactivity against recombinant protein under non-reducing conditions and has been independently confirmed by three groups (22, 68, 70, 72). In a single patient where serial serum samples were available, immunological remission (*i.e*., disappearance of anti-NELL1 antibodies) preceded clinical remission (22), consistent with what is seen in PLA2R-associated MN (19).

While initially felt to represent a mainly primary type of MN, NELL1-associated MN has been found to be associated with malignancy in 11.7-35% of cases (22). NELL1-associated MN was also reported in the setting of graft-*versus*-host disease (73) and in post-transplant nephrotic syndrome (72). A temporal association of lipoic acid use with NELL1-associated MN has also been shown in data from clinical trials, where remission of proteinuria occurred upon drug cessation (74).

### Protocadherin 7

A small subset of primary MN is associated with autoantibodies to protocadherin 7, and can be identified through immunohistochemical staining of kidney biopsies (31). PCDH7 is a transmembrane glycoprotein that is expressed at high levels in the nervous system (75), similar to THSD7A, NELL1, and SEMA3B, but has not been shown to be expressed by normal podocytes. Its presence in this form of MN was suggested based on LCM-MS. Similar to what had been shown for anti-PLA2R and anti-THSD7A, IgG reactive with recombinant PCDH7 could be eluted from the frozen tissue under non-reducing conditions demonstrating the presence of antibodies within the tissue in addition to enrichment of the antigen within glomerular immune deposits (31). Unique to this form of MN, PCDH7-associated MN has only low levels of complement staining on biopsy, a feature which might prompt the renal pathologist to further evaluate such a biopsy for PCDH7 positivity (31). The minimal C3 staining on biopsy was corroborated by MS, as spectral counts for many complement proteins (C3, C4A, C4B, C5 and C9) were reduced in PCDH7-associated MN when compared to a historical cohort of PLA2R-positive MN cases (76). IgG subclasses in PCDH7-associated MN were shown to be predominantly IgG1 and IgG4.

PCDH7-associated MN appears to be a disease of older adults (mean age = 61-66 years) and often presents with sub-nephrotic range proteinuria. PCDH7-associated MN was reported to account for 5.7-10.5% of PLA2R-, THSD7A-, EXT1/2-, SEMA3B-, and NELL1-negative MN cases (31). There are no known clinical associations to date and PCDH7 was not found to be localized to immune deposits in any cases of membranous lupus nephritis.

Further evidence of the rapidly developing field and consistent with Moore’s law are two new antigens identified and presented at abstracts at the American Society of Nephrology Kidney Week meeting in 2021. One antigen, protocadherin FAT1, may unravel the dilemma of *de novo* membranous nephropathy in the setting of stem cell transplantation or kidney transplantation. It was discovered by LCMD, followed by mass spectrometry. Similar to the other protocadherin protein associated with MN, PCDH7, there was minimal complement deposition see by immunofluorescence (77). The other newly identified autoantigen was Netrin G1 (NTNG1), identified by seroreactivity against human glomerular extract and subsequent western blotting (78).

We would like to emphasize that it is been becoming exceedingly difficult to extrapolate percentages to the larger MN population, since some studies have reported percentages of PLA2R-negative cases or triple/quadruple antigen-negative cases, while others have reported percentages of antigen-specific MN subclasses in their whole cohort. Therefore, we tried to provide our best estimates of the frequencies of each antigen in MN through adjusting to reported percentages of PLA2R MN (**Table 1**).

**Table 1:** Characteristics of the target antigens and biomarkers identified in membranous nephropathy.

Antigen	Size (kDa)	% Positivity (adjusted)	Compartment (transmembrane, secreted)	Podocyte expression	Circulating antibodies	Mean age (years)	Sex (M:F)	Distinctive associations
**PLA2R**	180	65-70%	Transmembrane	Yes	Yes	58	2-2.5:1	No distinctive associations
**THSD7A**	250	1.3-2.6%	Transmembrane	Yes	Yes	62	1.6:1	Malignancy 10%
**NELL1**	89	1.8-2%	Secreted	No	Yes	67	1.6:1	Malignancy 33%
**HTRA1**	51	1.4%	Secreted	Yes	Yes	67.3	1.3:1	No distinctive associations
**PCDH7**	116	1.0%	Transmembrane	No	Yes	61	1.3:1	Autoimmune disease 14%, Malignancy 21%
**SEMA3B**	83	<1%	Secreted	Yes	Yes	6.9 pediatrics, 36 adults	1.7:1	Pediatrics, Potential familial
**EXT1/2**	86/82	2.3 -3.4% (primary) and 17 -38.4% (MLN)	ER transmembrane	No/Yes	No	39.6	0.19:1	Membranous lupus nephritis
**NCAM1**	95	0.3-2% (primary) and 6.6% (MLN)	Transmembrane	Yes	Yes	34	0.4:1	Membranous lupus nephritis
**TGFBR3**	94	0 % (primary) and 5.5% (MLN)	Transmembrane	Yes	No	39.6	0.06:1	Membranous lupus nephritis

## Considerations for MN in Pediatric Patients

Compared to adult MN, identifying specific antigens has been more of an exception than the rule in children. Childhood MN has a lower overall rate of PLA2R-positivity of 45%, compared to greater than 70% in adults (79, 80). This frequency is quite low below the age of 10 but is significantly higher in adolescence. SEMA3B comprises approximately 10% of pediatric MN cases (30). Rarer considerations include MN in the setting of primary immunodeficiencies (81), antibodies to cationic bovine serum albumin (6), and anti-neutral endopeptidase in newborns (1). In children with immunodeficiency, MN is interestingly the leading cause of nephrotic syndrome. It is unclear why patients with impaired immunoglobulin production/hypogammaglobulinemia develop MN and the inciting autoantigen is unknown in most cases. PLA2R was found to be the culprit antigen in a neonate with immune dysregulation, polyendocrinopathy, enteropathy, X-linked (IPEX) syndrome (82).

In cationic BSA-induced MN, the dietary antigen cationic BSA (presumed to have arisen from modifications to bovine albumin present in infant formula) interacts with the anionic glomerular basement membrane and, with its corresponding antibodies, forms *in situ* immune complexes resulting in a loss of charge and size barrier, resulting in proteinuria (6). Complement pathway activation may also play a role in pathogenesis, resulting in the formation of membrane attack complex C5b-9 that localizes to subepithelial deposits. Infectious triggers should additionally be considered as potential etiologies in children, especially hepatitis B in areas with low rates of vaccination. Regardless, there is a large knowledge gap in our understanding of pediatric MN.

## Autoantigens in Membranous Lupus Nephritis

**Figure 3:**
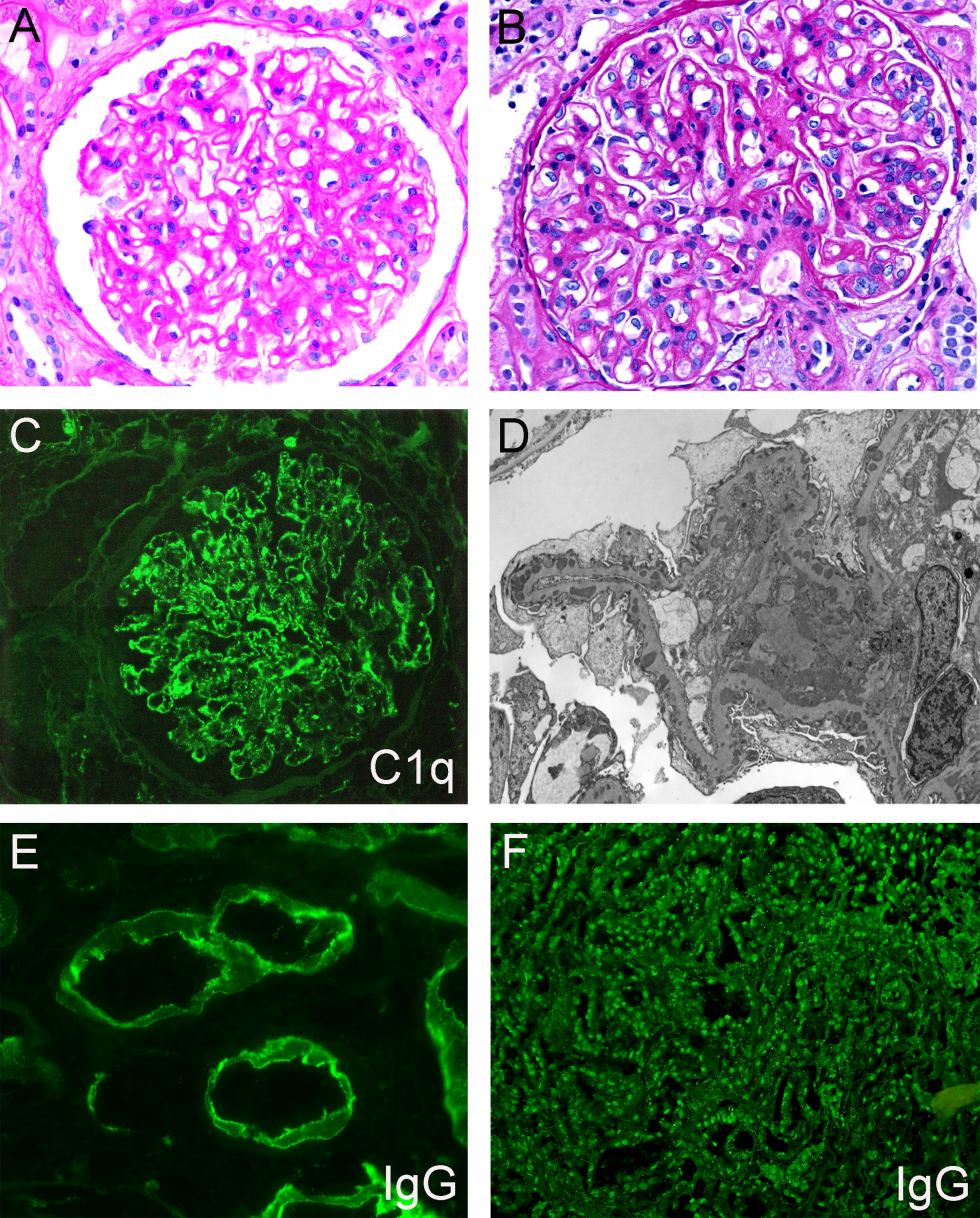
Representative histopathologic features of membranous lupus nephritis. **(A)** Glomerulus with mesangial expansion and prominent capillary loops, PAS, 400x. **(B)** Glomerulus with prominent capillary loops and endocapillary proliferation, PAS, 400x. **(C)** Granular mesangial and capillary loop staining, C1q immunofluorescence, 400x. **(D)** Electron photograph of subepithelial, intramembranous, and mesangial electron-dense deposits. **(E)** IgG immunofluorescence showing granular staining along tubular basement membranes, 600x. **(F)** IgG immunofluorescence demonstrating staining of tubular epithelial cell nuclei (‘tissue ANA’), 200x. Created with BioRender.com.

Membranous lupus nephritis (MLN, or class V LN) is the most common secondary etiology of MN and disproportionately affects young females of African or Hispanic descent, as well as other minority populations. There are some histopathologic findings on kidney biopsy that increase suspicion for MLN over primary MN (**Figure 3**). These include “full house” staining on biopsy (all three immunoglobulin heavy chains - IgA, IgG, IgM, and two complement components - C3, C1q), extraglomerular immune deposits (TBM staining, vascular deposits, or a ‘tissue ANA’ pattern of nuclear staining), subendothelial electron-dense deposits, and the presence of endothelial tubuloreticular inclusions. These findings have variable sensitivity and specificity alone, however when three or more of these five are present there is an 80% sensitivity and 95% specificity for a diagnosis of MLN over MN (83). Mesangial electron-dense deposits are also common and nearly always present in MLN, however, this finding has poor specificity as they can also be seen in primary MN cases. Additional, although non-specific, features suggestive of MLN are C1q positivity in conjunction with the presence of more than one IgG subclass (particularly IgG1 and IgG3) within glomerular immune deposits, consistent with activation of the classical complement pathway. In primary MN, IgG4 often predominates, which is uncommon in MLN. These histopathologic features are also shared in patients with MN secondary to autoimmune diseases other than systemic lupus erythematosus (SLE). Histopathologic features described in primary or MLN are shown in **Table 2**.

**Table 2:** Typical histopathologic features associated with each subtype of membranous nephropathy.

Antigen	Global or segmental	Proliferative changes	Predominant IgG subclass	IgA	IgM	C3	C1q	Mesangial	Subendothelial	TBM deposits
**PLA2R**	Global	No	IgG4	10%	15%	91%	2%	10%	1%	Absent
**THSD7A**	Global	No	IgG4	40%	10%	80%	10%	40%	10%	Absent
**NELL1**	Segmental or Global	No	IgG1	8%	10%	78%	0%	24%	0%	Rare
**HTRA1**	Segmental or Global	No	IgG4	0%	0%	100%	7%	14%	0%	Absent
**PCDH7**	Global	No	IgG4/IgG1	7%	0%	43%	29%	0%	0%	Absent
**SEMA3B**	Global	No	IgG1	9%	18%	91%	45%	0%	0%	Present
**EXT1/2**	Segmental or Global	Yes (27%)	IgG1>IgG2>IgG3>IgG4	49%	46%	94%	48%	99%	20%	Present
**NCAM1**	Segmental or Global	Yes (25%)	IgG1>IgG2>IgG3-IgG4	65%	68%	85%	55%	95%	55%	Present
**TGFBR3**	Segmental or Global	Yes (29%)	All IgG subclasses	71%	88%	88%	59%	94%	35%	Present

While the antigenic targets involved in the majority of primary MN cases have been discovered, the majority of autoantigens in MLN are unknown. To date, three autoantigens have been described in membranous lupus nephritis, which together comprise approximately 1/3 of all MLN cases. These include the exostosin 1/exostosin 2 complex (EXT1/2) (21), neural cell adhesion-molecule 1 (NCAM1) (32), and transforming growth factor β receptor 3 (TGFBR3) (33). PLA2R positivity can be rarely seen in MLN (in up to 5.3% of cases) (84). Contactin 1 (CNTN1) is also described in the setting of autoimmunity, although not specifically within membranous lupus nephritis.

### Exostosin 1/2 (EXT1/EXT2)

The first biomarker to be described in MLN is the EXT1/EXT2 complex, identified by LCM-MS of kidney biopsies. The EXT1/EXT2 complex represents a hetero-oligomeric glycosyltransferase that requires both proteins for enzymatic function. It participates in GBM homeostasis through regulation of heparan sulfate (85). EXT1 mutant alleles have been previously described in familial kidney disease, with loss of function mutations leading to proteinuria (86).

Both the EXT1 and EXT2 components of the heterodimer are present within deposits in all cases and therefore staining for either component can be performed to identify positive cases. EXT1/EXT2-positive MN appears tightly associated with autoimmunity, with 70% of patients having a positive ANA, 35% having a clinical diagnosis of SLE and 12% of patients having mixed connective tissue disease at the time of biopsy (21). The frequency of EXT1/EXT2 positivity in MLN is 17.0-38.4% (33, 87, 88). From the largest cohort of 374 patients with MLN, 32.6% had EXT1/EXT2-positivity. Of these patients, 75% were ‘pure’ MLN cases (ISN/RPS class V) and 25% had a concurrent proliferative lupus nephritis component in addition to MLN (ISN/RPS class III/IV + V) (87). Confocal microscopy studies have confirmed the presence of EXT1/EXT2 immune complexes along the subepithelial surface of the GBM and have demonstrated co-localization with IgG. While accumulation of this protein occurs in the subepithelial space, no autoantibodies were identified within serum under reducing or non-reducing conditions, questioning whether this represented a true autoantigen or may merely represent a biomarker of disease (21).

Although unclear at this time whether the EXT1/EXT2 complex is a putative autoantigen or a biomarker, the finding has prognostic significance. Two independent investigations indicated its value as a biomarker of favorable outcome with less progression of kidney disease in patients with MLN, when compared to EXT1/EXT2-negative MLN (87, 88). Remarkably, these studies also included patients with a concurrent proliferative component and showed a similar prognostic significance to EXT1/EXT2 status.

### Neural Cell Adhesion Molecule 1

NCAM1 is an immunoglobulin superfamily cell surface glycoprotein that is expressed within the central nervous system, immune system, and within podocytes (89, 90). It is a negative regulator of the expansion of T cells and dendritic cells in the adaptive immune response (91). NCAM1 was identified to be an autoantigen in membranous lupus nephritis through tissue-based proteomic studies, utilizing both LCM and tissue IP (32). NCAM1 co-localizes with IgG within glomerular immune deposits and is present in 6.6% of all MLN biopsies and 0.4-2.0% of primary MN cases (45). Of patients with MLN, 25% had concurrent class III or class IV lupus nephritis (32). Seroreactivity was identified by immunoblotting with NCAM1 recombinant protein exclusively under non-reducing conditions and by indirect immunofluorescence (IFA) using a NCAM1-overexpressing cell line.

Patients with NCAM1-associated MN were predominantly female (70%), with a mean age of 34 ± 12.1 years. Interestingly, 40% of NCAM1-associated MN patients with SLE had an increased frequency of neuropsychiatric manifestations at the time of biopsy (8/20 patients), a rate 4-5 times higher than reported in SLE overall (9% prevalence). There are limited data to conclude whether NCAM1 may be a link between nephritis and neuropsychiatric disease in patients with SLE, but the question warrants further investigation to determine if such a connection exists. Manifestations of SLE vary in time and it could be helpful to determine if patients with NCAM1 autoantibodies are at future risk for neuropsychiatric disease and therefore might benefit from closer monitoring.

## TGFBR3

The type III transforming growth factor-beta receptor (TGFBR3) was identified as a putative target in MLN by tissue-based proteomics utilizing LCM and tissue IP (33). TGFBR3 is an accessory receptor for TGF-β signaling and is involved in negative regulation of T-cell dependent antibody responses through reducing CD4^+^ T-cell specification to the Th1 lineage (92). TGFBR3 is expressed within podocytes.

TGFBR3 is positive within 5.5% of MLN biopsies and is not identified in primary MN cases (33). Patients with TGFBR3-associated MN had a mean age of 39.6 ± 16.1 years and were predominantly female. Nearly all patients had a history of autoimmune disease with 82% having a diagnosis of systemic lupus erythematosus (SLE) at the time of MN biopsy (33). A concurrent proliferative (class III or IV) component was identified in 29.4% of cases. Several different methods were tried but failed to show seroreactivity against TGFBR3, including immunoblotting patient sera against the recombinant protein, use of a TGFBR3-overexpressing cell line in a cell-based indirect immunofluorescence assay, and immunoprecipitation with human glomerular extract. As circulating antibodies have not yet been identified, we cannot determine whether TGFBR3 represents an autoantigen or a biomarker for MLN, similar to the situation with EXT1/EXT2 (21, 33).

Moreover, it conceivable that, despite the paradigm that immune complexes in lupus form extra-renally in the circulation, the pathogenesis of pure lupus nephritis may be more similar to that of primary MN with antibodies targeting intrinsic, induced, or planted podocyte antigens to form immune complexes *in situ*, an argument that is supported by the lack of mesangial or subendothelial deposits in some cases of MLN.

## Additional Autoimmune Diseases Associated With MN

In addition to SLE, other autoimmune diseases have been associated with MN, including sarcoidosis (38, 93, 94), urticarial vasculitis (38, 95), ANCA-associated glomerulonephritis (38, 96), rheumatoid arthritis (38, 97), Sjogren syndrome (38, 98, 99), systemic sclerosis (38, 100, 101), thyroiditis (38, 102, 103), chronic inflammatory demyelinating polyneuropathy (CIDP) (104–106), autoimmune myositis (99, 106), and ankylosing spondylitis (38). Therefore, patients with a positive ANA, systemic manifestations, and MN should undergo a thorough workup to exclude autoimmune disease.

Myeloperoxidase (MPO) is a target autoantigen associated with MN in the setting of ANCA-associated disease. A concurrent crescentic glomerulonephritis is frequent, but MN can occur in its absence. These cases are PLA2R-negative and MPO has been demonstrated in the epimembranous immune deposits in glomeruli (96, 107, 108).

Membranous-like glomerulopathy with masked IgG kappa deposits (MGMID) is an autoimmune disease that can exhibit a membranous pattern on kidney biopsy and affects young women, often with positive antinuclear antibodies (109). In most cases, IgG deposits along the glomerular capillary loops are ‘masked’ meaning that they are not identified by routine immunofluorescence but are seen by paraffin immunofluorescence after pronase digestion of FFPE tissue. Evaluation by immunofluorescence after pronase digestion is triggered when there are subepithelial electron-dense deposits by electron microscopy, but no IgG staining on routine immunofluorescence microscopy (109, 110). This glomerular disease looks like membranous nephropathy on a kidney biopsy, but is a separate disease entity, of which can be identified by staining for the biomarker serum amyloid P (SAP) (29). It should be considered in the differential diagnosis when MN has kappa restriction (more specifically, IgG1-kappa restriction). Although light chain restricted, it is not thought to be a paraprotein-mediated disease and instead the antigen SAP interacts with IgG1-kappa (111). Conversely, other forms of MN that are light chain restricted should be investigated for an underlying lymphoproliferative disorder, particularly if PLA2R negative (in 75% of cases) (112).

CNTN1 was recently identified as a target antigen in MN with chronic inflammatory demyelinating polyneuropathy (CIDP) and autoimmune myositis (105). It is a cell adhesion molecule near the node of Ranvier of neurons. Anti-CNTN1 autoantibodies can be monitored by ELISA-based testing (104). Patients with autoimmune neuropathy that is CNTN1-associated have an increased prevalence of nephrotic syndrome, which is often found to be due to MN. Antibodies were IgG4-subclass predominant (106). LCMD of glomeruli was performed and confirmed the presence of CNTN1 in these patients. Therefore, CNTN1 may represent a link between autoimmune neuropathy and nephrotic syndrome. Very rare cases of CNTN1-positive MN have also been identified in patients without neurological symptoms (106).

## Membranous Antigens Are Proteins Shared by the Central Nervous System

Perhaps relevant to these links to neuropsychiatric disease or autoimmune neuropathies, several MN autoantigens are proteins expressed in both neurons and podocytes. There are similarities in signaling between podocytes and neurons, both of which are specialized cells with arborized processes supported by robust cytoskeletal dynamics and an intercellular communication utilizing similar proteins expressed at synapses and foot processes, such as synaptopodin and dendrin (113, 114). Other proteins with restricted expression within podocytes and neurons include neurexin 1, NPHS1, SYNPO, and KHDRBS3, among others (113, 114). Proteins required for axonal extension and survival in neurons, polarity, and preservation of synaptic connections are also expressed within podocytes and are crucial to preserve the glomerular filtration barrier. This link has recently culminated in identifying CNTN1 as the main antigen involved in nephrotic syndrome in those with demyelinating neuropathy (106). An additional potential target for CIDP-related MN is neurofascin 155 (NF155) (104), a similar paranodal protein. Other target antigens in MN expressed in neurons include THSD7A (115), NCAM1 (116, 117), NELL1 (118), HTRA1 (119), PCDH7 (120), and SEMA3B (121).

## Sources of Potential Membranous Autoantigens

Antigens targeted in MN can be intrinsic podocyte proteins, podocyte proteins that may be induced by certain stressors, extrinsic non-podocyte proteins that become planted in a subepithelial position, or those that precipitate from circulating immune complexes. The concept that target antigens in MN are often podocyte-expressed proteins is relatively recent. The antigenic component of rat proximal tubular brush border is megalin which, in a not-well-understood quirk of nature, is universally expressed by the podocyte in the rat, but not so in other species. PLA2R and THSD7A were identified as target antigens in MN before they were found to be podocyte proteins. Detailed scrutiny of the more-recently identified antigens may reveal that they are expressed by podocytes in some cases, including HTRA1, SEMA3B, NCAM1, and TGFBR3, but not in others, including EXT1/2, NELL1, PCDH7, and CNTN1. However, our knowledge of what is or is not expressed by normal human podocytes in the resting state is not comprehensive, and new studies consistently reveal novel podocyte proteins. There may also be age-related developmental changes in expression, or induced expression by disease or environmental factors. Such an induced expression may, for instance, explain the segmental pattern of NELL1-associated MN, since NELL1 does not seem to be normally expressed by the podocyte. In challenging the prevailing paradigm in understanding MN, the presence of antigens not inherently expressed in glomeruli, such as EXT1/2, NELL1, and PCDH7, may suggest more than one pathogenic pathway can lead to similar pathologic manifestations.

## Medications Inducing Membranous Nephropathy

Multiple medications are described to cause MN, comprising 6.6-14% of total MN in prior studies (122–124). Drug-induced MN was reported with therapy with gold salts, penicillamine, bucillamine, captopril, non-steroidal anti-inflammatory drugs (NSAIDs), selective COX-2 inhibitors, tiopronin, trimethadione, and lipoic acid (74, 123). NSAIDs are currently the leading offender and this form of MN is often reversible, remitting with discontinuation of the offending drug (125). Some medications associated with development of MN may act as weak reducing agents and potentially modify protein folding. Alternatively, they may serve as haptens.

## Infectious Triggers of Membranous Nephropathy

Some infections have been identified in association with MN and in certain cases the microbial protein has been identified within immune complexes. It is unclear whether microbial proteins are true antigenic targets or are passively trapped within the subepithelial space. Hepatitis B virus (HBV) infection can induce MN with the hepatitis B e antigen (HBeAg) identified within subepithelial deposits. Proteinuria persists with HBV antigenemia and one-third develop progressive disease, despite treatment (126, 127). In a recent study, the prognosis of HBV-associated MN was similar to that of PLA2R-positive MN (128). The incidence of HBV-associated MN may be even higher in children, as children with nephrotic syndrome in endemic regions commonly have HBV-associated MN (129). HBV-associated MN has been described in the setting of PLA2R and THSD7A positivity as well, which could be coincidental or instead represent a viral trigger for autoimmunity (130).

Hepatitis C virus (HCV) infection has also been reported as a secondary cause of MN which can remit with antiviral therapy (131, 132). Membranous nephropathy can also be associated with human immunodeficiency virus (HIV) infection; in one study, 9% of HIV-infected patients with proteinuria had MN (133, 134). Syphilis, which can be co-morbid with HCV or HIV infection, is also associated with MN. Treponemal antigens have been identified within glomerular immune complexes (135) with resolution of nephrotic syndrome following treatment of the syphilis (136). Schistosomiasis can also trigger MN in endemic regions (137, 138). Elution of immune complexes from kidneys of patients with schistosomiasis identified reactivity against *Schistosoma mansoni* by indirect immunofluorescence (139). Recently in the COVID-19 pandemic, cases of MN have been reported in patients with SARS-CoV-2 infection, or post-SARS-CoV2 vaccination (140–146). There is an increased frequency of PLA2R-negative MN than expected for primary MN, however it is unclear whether this is due to molecular mimicry with a viral component, such as the spike protein. It is possible that PLA2R-positive MN may be more likely to be empirically treated without a biopsy in those with positive serology, particularly during the COVID-19 pandemic where elective procedures which include kidney biopsies have been halted during disease surges.

It is intriguing that only a few infections trigger MN. This could be due to molecular mimicry with a limited subset of microbial proteins found in these associated infections that might induce autoreactivity to host proteins. A peptide contained within the dominant epitope in the N-terminal cysteine-rich domain of PLA2R also exists in D-alanyl-D alanine carboxypeptidase, a cell wall enzyme in several bacterial strains (147). No clinical evidence has yet been produced to support this potential connection. Molecular mimicry may also play a role in the case of HTRA1-associated MN, in which bacterial form of the HTRA protein found in the cell wall of *Orientia tsutsugamushi* is known to be antigenic and has been used in a vaccine developed against scrub typhus, demonstrating immunogenicity (148).

Toll-like receptor responses may also be involved through interacting with microbial nucleic acids. Interferon regulatory factor 4 (IRF4), a negative regulator of TLR signaling, was identified in a large genome wide association study for MN as a highly significant risk locus (149). IRF4 binds MyD88 (a common signal transduction protein critical to TLR signaling) and competes with IRF5. In murine lupus models, induction of ligands to active TLR3, TLR7, and TLR9 (which interact with dsRNA, ssRNA, and CpG DNA respectively) increase severity of nephritis (150).

## Malignancy-Associated Membranous Nephropathy

Another secondary etiology of MN is hematologic or solid organ malignancy, comprising approximately 10% of MN cases. MN is the leading cause of nephrotic syndrome in patients with malignancy and patients with MN have a three-fold increase in the incidence of cancer compared to the general population (151, 152). Therefore, cancer screening is an initial step after MN diagnosis (153). Concurrent MN and malignancy confer a poor clinical outcome with a reduced rate of remission and a four-fold increased risk of thromboembolic disease (153). When MN is malignancy-associated, proteinuria can respond dramatically to resection, chemoradiation, or other treatments. Likewise, proteinuria may worsen with recurrence or metastasis.

Because both MN and malignancies tend to occur in older individuals, it can often be difficult to determine if the cancer is causal or coincidental (39). To classify the MN as malignancy-associated, three key factors may be present, although all may not necessarily be known at the time of diagnosis. The patient’s diagnosis of MN and cancer should be temporally associated, proteinuria should resolve with cancer remission, and/or relapse of the malignancy causes recurrence of the MN. Multiple tumor types were identified in temporal association with membranous nephropathy, including carcinomas (58), soft tissue tumors (154), melanoma (155), thymoma (154), and lymphoma (68, 156, 157). Histopathologic clues for malignancy-associated MN include PLA2R-negativity, a segmental pattern on IgG staining, endocapillary hypercellularity (154), and IgG1 and IgG2-predominant immune deposits (as most cases of primary MN have IgG4-predominant immune deposits) (158). NELL1 is the autoantigen in approximately one-third of malignancy-associated MN cases while THSD7A is found in approximately 10% (68). Both NELL1 and THSD7A have been shown to exhibit increased protein expression in certain human cancers (59, 64, 159).

The mechanisms underlying malignancy-associated MN are largely unknown. It is possible that dysregulated expression of immunogenic proteins by the tumor incites autoimmunity with subsequent deposition of immune complexes within the subepithelial space. Genetic mutations within the tumor may alter the amino acid sequence to create neo-epitopes that are immunogenic. Alternatively, there could be molecular mimicry of a tumor antigen that shows sequence similarity to a podocyte protein (39).

Increased expression within the tumor due to copy number increases, as has been seen with THSD7A due to polysomy of chromosome 7 (58), may also occur for other autoantigens. Many cancers carry a high mutational burden (160), particularly those with mismatch repair defects, of which could create neo-epitopes that may elicit an autoimmune response. Increased RNA transcript stability, protein stability, or epigenetic modifications are other possibilities. Some tumors have a high frequency of over-expression of a target protein, as seen in both THSD7A and NELL1, yet a majority of patients with malignancy do not develop kidney disease. It is possible that a second hit may be required, which warrants further investigation.

## Antigen-Based Classification of MN – Abandoning Primary *Versus* Secondary

The categorization of MN as either primary or secondary was based on the absence or presence of a detectable underlying cause, such as systemic autoimmune disease, cancer, or infection, as discussed above. Subsequently, after the identification of PLA2R as target antigen in the majority of primary MN, PLA2R-negative cases elicited an extensive workup to identify a possible secondary etiology (37). Recently identified autoantigens have been detected, commonly or more rarely, in settings that would more typically be associated with a potential secondary etiology of the MN, calling into question whether MN associated with a known antigen is necessarily primary. Due to the inability to assign a particular target antigen as exclusively representative of primary or secondary disease, the field has been moving toward a more antigen-based classification system (45).

An overview of demographic, clinical, and histopathologic characteristics for the MN subtypes associated with each of these various antigens in MN is provided in **Tables 1**, **2**. Given the large number of autoantigens identified to date and extrapolating from clinical experiences with PLA2R and THSD7A, such an antigen-based classification system could result in development of precision diagnostics for monitoring disease and has been advocated by others (45, 161). The large number of MN antigens thus far identified has also made it impractical to perform antigen subtyping by immunostaining of the kidney biopsy for each one. Instead, a mass spectrometric approach might instead be considered in cases of PLA2R-negative MN, similar to what is done for the typing of amyloidosis cases that are found to be non-AL amyloid-associated (161).

**Figure 4:**
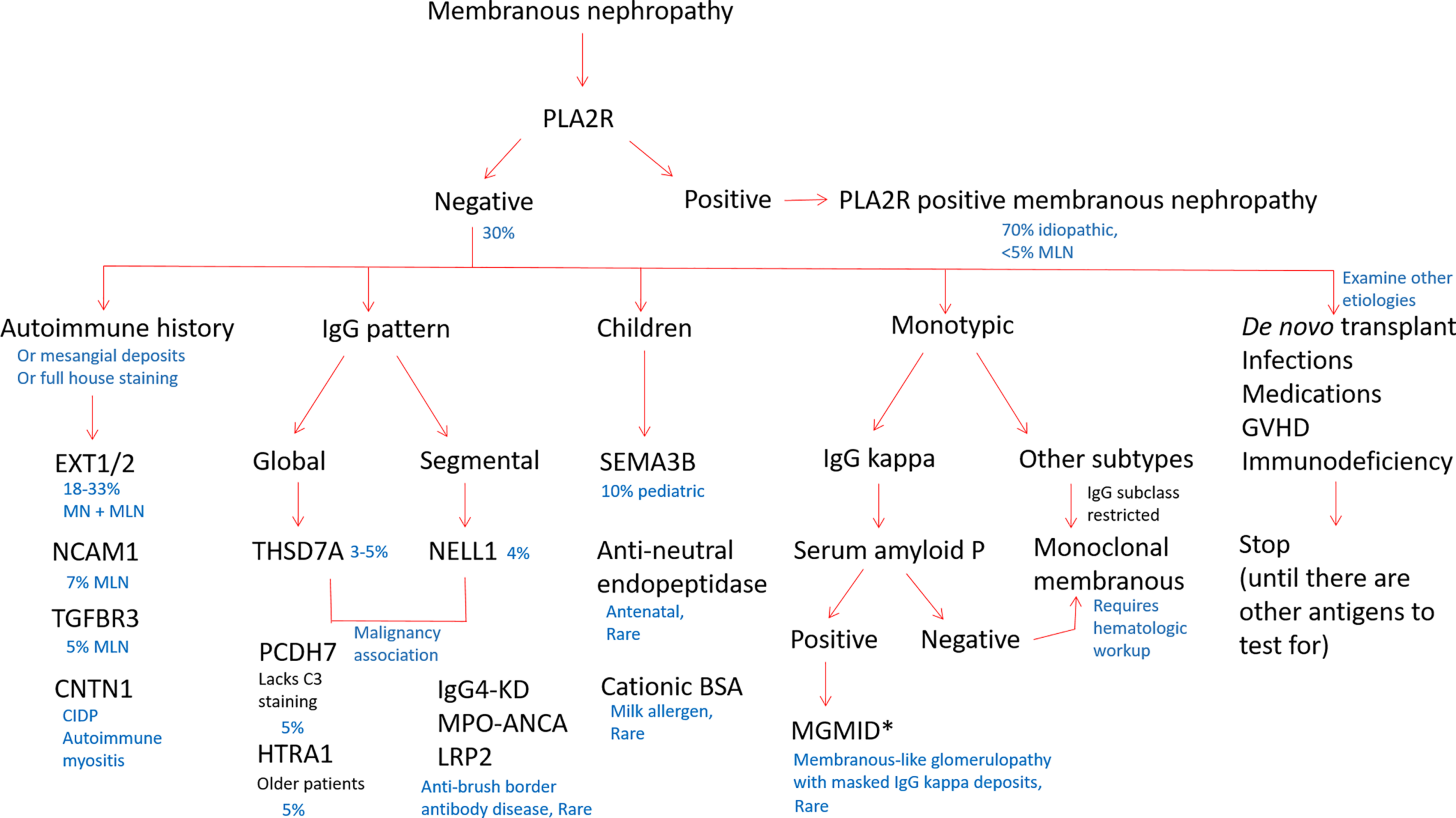
Proposed staining algorithm for phenotyping of membranous nephropathy cases. For ‘all comers’ of MN cases without a known history of systemic lupus erythematosus, we suggest staining for PLA2R, as it will identify the majority of cases (approximately 70%). For biopsies that are PLA2R negative, the clinical history, demographics, and pattern of immune reactants on biopsy could guide which antigens to evaluate. For patients with an autoimmune history (positive ANA or history of autoimmune disease), staining for EXT1/2, TGFBR3, NCAM1, and CNTN1 can together identify approximately 40% of MN cases secondary to autoimmune disease or membranous lupus nephritis. Children with MN are most commonly PLA2R-positive as adults, although SEMA3B staining will pick up approximately 10% of pediatric MN cases. In neonates, anti-neutral endopeptidase and cationic BSA are additional considerations. NELL1 and THSD7A may be enriched in patients with malignancy. The IgG pattern on biopsy is useful to choose additional antigens for staining, as THSD7A, PCDH7, and HTRA1 typically have a diffuse and global granular capillary loop pattern and NELL1 MN often shows segmental IgG staining. IgG4-related kidney disease, ANCA-associated glomerulonephritis (p-ANCA/MPO antibodies), and LRP2-associated nephropathy also often show a segmental IgG pattern along capillary loops. When MN is restricted to one light chain, cases with IgG kappa can be evaluated for SAP to identify membranous-like glomerulopathy with masked IgG kappa deposits (MGMID)*. If lambda light chain restricted or negative for SAP, IgG subclasses are helpful to identify if the MN is restricted to one subtype, for which a hematologic workup can be indicated to evaluate for an underlying lymphoproliferative disorder as a driver of disease. In patients with *de novo* MN following transplantation, infections, medications, graft-*versus* host disease, or immunodeficiency, it is common to not identify a known autoantigen at this time. *In the setting of subepithelial electron-dense deposits by electron microscopy, but no IgG staining by routine immunofluorescence microscopy, pronase digestion of FFPE tissue can ‘unmask’ immune deposits in MGMID and is required in the majority of cases. Created with BioRender.com.

However, as such an MS-based strategy for the typing of MN is currently impractical for routine clinical practice, we propose a staining algorithm for antigen subtyping based on clinical and pathologic features (**Figure 4**). As PLA2R-positive MN comprises the majority of MN cases, PLA2R staining should be performed first as a high-yield diagnostic assay. For PLA2R-negative cases, clinical considerations should be utilized to inform further staining. For patients with known autoimmune disease, or those with positive antinuclear autoantibodies that lack a diagnosis of a particular rheumatologic condition, EXT1/2, NCAM1, and TGFBR3 staining can identify approximately 40% of cases, although our current knowledge gap of the remaining antigens still leaves the majority of SLE patients untypeable. Age can be a useful factor, as elderly individuals may have an increased frequency of HTRA1- or PCDH7-associated MN (23) and young children have an enrichment in SEMA3B positivity (30). Histopathologic variables can be informative, including low complement staining enriching cases of PCDH7-associated MN, segmental or incomplete capillary loop staining in NELL1-positive cases or patients with concurrent ANCA-associated glomerulonephritis (71).

## How Do Antibodies Cause Proteinuria?

Subepithelial immune complex formation is the hallmark of MN. Complement pathway activation has been considered as the paradigmatic mechanism leading to podocyte injury and failure of the glomerular filtration barrier, resulting in the nephrotic syndrome. However, other mechanisms inducing podocyte injury have been postulated and the primacy of complement as a pathomechanism in MN has been challenged. In the passive Heymann nephritis model, proteinuria depends on activation of the complement system with subsequent formation of the membrane attack complex (C5b-9) (162, 163). Other studies, however, provide evidence that proteinuria can develop in the absence of certain complement components (164, 165). For example, in a mouse model of MN with passive transfer of human anti-THSD7A antibodies, proteinuria developed without C3 or C5b-9 deposits.

It is possible that any antigen-antibody complex at the appropriate location can activate complement and thereby induce MN. The immune deposits in human MN contain substantial amounts of complement, including C3 and C5 cleavage products and the terminal C5b-9 complex, yet the predominant IgG subclass in primary MN is IgG4, which is incapable of activating complement *via* the classical pathway (166). In addition, mass spectrometry of LCM-dissected glomeruli revealed increased protein expression of complement components C3, C4, C5, C6, C7, C8, and C9, as well as regulators of complement pathway activation (76). The absence of C1q in subepithelial deposits suggested that either the alternative pathway or mannose binding lectin (MBL) pathway are involved in disease pathogenesis. The presence of C4 in the absence of C1q suggests a role for MBL pathway activation. However, reported cases of MN in patients deficient in MBL (167) and ficolin 3 (168) suggest involvement of alternative complement pathway. Recent studies using the IgG4 subclass of anti-PLA2R have provided *in vitro* evidence of direct cytotoxic effects on podocytes *via* the MBL pathway of complement activation (169).

Additionally, antibodies may induce proteinuria by functional interference with proteins critically involved with maintaining the glomerular filtration barrier or podocyte health. Such a mechanism was suggested by the impairment of cellular adhesion to type IV collagen, a key matrix molecule in the GBM, by PLA2R autoantibodies (170), although other studies did not confirm this (171). Further evidence gleaned from a mouse model of MN suggested that THSD7A autoantibodies may directly lead to cytoskeletal structural alterations that result in proteinuria (172) possibly related to the fact that THSD7A localizes directly beneath the slit diaphragm (173). HTRA1 is involved in extracellular matrix (ECM) homeostasis (90), but whether anti-HTRA1 antibodies interfere with podocyte-ECM cross-talk has yet to be determined.

A prerequisite for circulating antibodies to interact with a target podocyte antigen is the presence of one or more exposed humoral epitope in an extracellular location, either within the extracellular region of transmembrane protein or on a secreted protein that associates with the podocyte basal surface or the GBM. While many epitopes likely exist in accessible sites dictated by the native conformation of the protein, others may be cryptic and require additional exposures or modifications, as has been suggested for SEMA3B. Both THSD7A and PLA2R proteins are transmembrane proteins with extracellular domains that are exposed and accessible to the humoral immune system. HTRA1 is a matrix modifying enzyme that is secreted in conjunction with extracellular matrix molecules that serve a substrate for its protease activity and thus can localize to the GBM and be targeted by autoantibodies in this environment (174–177).

Certain intracellular proteins, such as superoxide dismutase, can be induced by oxidative stress by podocytes or other nearby cells such as glomerular endothelial cells and may be accessible as target antigens at the cell surface, subepithelial space, or associated with the GBM (41, 178, 179). Prunotto et al. showed the presence of IgG4-dominant antibodies against endothelial proteins superoxide dismutase 2 (SOD2), aldose reductase, and enolase in MN (179). The recent discovery of non-podocyte proteins as MN antigens, including PCDH7, NELL1, and EXT1/2, further established that non-podocyte proteins can indeed be MN antigens. Moreover, the identification of microbial proteins and tumor proteins within immune complexes supports the concept that non-native podocyte proteins can be targeted antigens. The pathophysiologic mechanisms through which antibodies targeting antigens not expressed in podocytes remain to be fully elucidated. One may argue that antigens that become planted in a subepithelial position by virtue of charge or other characteristics are also considered *in situ* immune complex formation and does not necessarily indicate that the antigen is an intrinsic podocyte protein.

Understanding how these non-native proteins become planted can help us understand disease pathogenesis. Treatment of podocytes, which have low baseline expression of these intracellular proteins, with hydrogen peroxide to induce oxidative stress resulted in SOD2 expression along the outer plasma membranes of podocytes (179). This suggests that some proteins may be induced as neoantigens under oxidative stress. It was postulated the antigenicity starts at the podocyte level with neo-epitope exposure triggering autoimmunity, with subsequent *in situ* immune complex formation and nephrotic syndrome (179–182).

Some environmental factors may also be involved in triggering autoimmune responses that may occur outside of the kidney. MN is more common in areas with high levels of microparticulate (PM2.5) air pollution, potentially implicating this environmental exposure as a risk factor for disease initiation (183). This has shed light on a potential extrarenal site of antigenicity, as PLA2R is known to be expressed within alveolar macrophages of the lung and may be more highly expressed in the setting of airway inflammation (184). It is possible that such induced protein overexpression overwhelms the cell’s capacity to properly translate and fold these highly-disulfide bonded proteins, resulting in misfolding, perturbed protein trafficking, or endoplasmic reticulum stress that could ultimately trigger an immune response in genetically susceptible individuals. Extrinsic protein overexpression as a trigger for antigenicity is also suggested by malignancy-associated THSD7A-positive MN in which THSD7A is abnormally expressed in tumor tissue due to polysomy of chromosome 7, providing a potential source of antigen that can overwhelm host tolerance mechanisms (59).

## Genetic Factors Predisposing to Membranous Nephropathy

Genetic predilection has also been implicated in MN pathogenesis. Genome-wide association studies (GWAS) and whole exome sequencing (WES) have revealed highly significant risk alleles predisposing to MN. A single-nucleotide polymorphism (SNP) at the *HLA-DQA1* locus has been associated with higher risk of developing PLA2R-associated MN (185), more so if concurrent with a SNP in *PLA2R1* (185). Most SNPs in *PLA2R1* that associate with disease risk are common in the normal population (186, 187), so it is unclear whether there is multi-hit hypothesis leading to development of MN in only those with particular HLA haplotypes, other genetic aberrations, or exposure to environmental factors. It is possible that the *PLA2R1* SNP drives abnormal expression of PLA2R, while the HLA SNP more easily allows presentation of PLA2R-derived peptides in the binding grooves of major histocompatibility complex class II molecules. The most significant SNPs in *PLA2R1* are intronic and thus are not expected to change protein structure (50, 188, 189), but rather may alter protein expression level with consequent misfolding and disturbed trafficking leading to antibody formation. The presence of different transcript isoforms or altered expression levels corresponding to the specific antigen remains to be investigated.

## Epitope Spreading

Epitope spreading is an immunologic phenomenon whereby an initial antibody response to a given antigen may extend from one particular location (epitope) on the antigen to involve other region(s) of the same antigen (intramolecular spreading) or nearby or related antigens (intermolecular spreading) (6). Epitope spreading is a common phenomenon in autoimmune disease (190–193). Initial experimental evidence that epitope spreading occurs in MN and affects its severity was demonstrated in Heymann nephritis using only N-terminal domains of megalin to trigger subsequent humoral responses to more distal portions of the molecule (7). Epitope spreading was subsequently described in humans with PLA2R-associated MN. In PLA2R-associated MN, patients first appear to develop autoreactivity against an epitope within the cysteine-rich domain (CysR). Epitope spreading to include the CTLD1 and CTLD7 domains (in addition to the CysR) is associated with poor prognosis (55). Some studies suggested that in patients with PLA2R-positive MN, epitope spreading during follow-up associated with disease progression, whereas reverse spreading back to only a CysR profile associated with favorable outcomes (174, 194). However, this finding has been debated by other studies, which suggest that the correlation with epitope spreading and worse prognosis is more related to overall PLA2R antibody titers (195–197) and it remains unclear whether this truly reflected spreading or concurrently target epitopes from disease initiation.

A better understanding of the evolution and repertoire involved in epitope spreading to any MN target antigen may help to guide prognosis, to understand the initial triggering events in disease pathogenesis, and to allow development of antigen-targeted therapies. While epitope spreading is important in the initiation and progression of MN and other autoimmune diseases (198–201) and carries potential prognostic value, it is not yet utilized in routine clinical practice.

Intramolecular epitope spreading, as described above, may be more common than intermolecular epitope spreading. Rare reports of dual positivity for antigenic targets has been observed with concurrent PLA2R and THSD7A-positive cases (202), PLA2R and EXT1/2 co-positivity (203), THSD7A and EXT1/2 co-positivity (203), and dual NCAM1 and EXT1/2 staining (32). However, no study has shown that one autoimmune reaction predated the other, so the existence of intermolecular epitope spreading (*i.e.*, antibody-mediated cytotoxicity due to one antigen resulting in exposure of the second protein to the immune system) is only speculative. The incidence of PLA2R and THSD7A dual positivity is approximately 1%, shown in a study where 2/258 cases of primary MN shown dual positivity by immunostaining and serologic testing (202). Insufficient data exists to determine whether dual positive cases are associated with a worse prognosis.

In contrast to these infrequently-encountered dual responses, most antigen-based MN subtypes are mutually exclusive. It is unclear why, in the majority of cases, there is an autoimmune response to only a single antigen. One possibility is related to an individual’s class II HLA repertoire and the decreased likelihood of possessing two or more HLA risk alleles that could support initiation and propagation of an autoimmune response to both antigens. If intermolecular epitope spreading were related only to cell damage, we would expect more cases of dual reactivity due to the universal expression of PLA2R and THSD7A by the human podocyte.

## Serologic Monitoring of Disease

One of the most evident benefits of knowing the antigenic target of a humoral immune response is the ability to detect and measure circulating autoantibodies. Serologic testing (or “immune surveillance”) offers the ability to monitor immunologic disease activity and response to therapy in a non-invasive manner. As demonstrated with anti-PLA2R, autoantibody levels correlate with disease activity but with a lag time, decreasing and often disappearing prior to a full proteinuric response with treatment, or increasing after remission to signal oncoming relapse of disease. As serologic remission precedes clinical remission, it can be useful to adjust immunosuppressive therapy accordingly and can inform prognosis. Serologic diagnostic tests for PLA2R-associated MN (51) and THSD7A antibodies (61) show a high sensitivity (70.6-78%) and specificity (91-94.6%) (52, 53), although are subject to false negatives or false positives if used to screen all patients with nephrotic syndrome.

As a proof of concept and extrapolating from the PLA2R experience, a combination of IFA and ELISA-based testing provided high specificity for disease. This has allowed providers in some situations to avoid the need to perform a kidney biopsy in patients who have with anti-PLA2R antibodies and rather to monitor them according to anti-PLA2R titers (204, 205). This diagnostic strategy is now supported in the 2021 KDIGO Clinical Practice Guideline for the Management of Glomerular Diseases in not mandating a kidney biopsy to confirm PLA2R-associated MN in a seropositive patient (206). This is supported by our experience in which we see a lower frequency of PLA2R-positive kidney biopsies in our recent cohort, compared to historical studies suggesting that clinicians are already deciding not to biopsy patients diagnosed by the non-invasive serological test (33).

The identification of new antigens in MN may also provide a means for clinical monitoring when circulating antibodies are detectable, yet there is a need for development of validated and reproducible immunoassays directed towards these antigens for use in the clinical setting. With such advances in serologic testing, the diagnosis and monitoring of the recently identified MN subtypes may be possible and more widely available. However, some subtypes of MN, including EXT1/2- and TGFBR3-associated disease, have yet to have autoantibodies identified, creating a challenge for development of these assays. Identifying autoantibodies directed against all distinct MN types could alter treatment paradigms for MN by adding to the list of treatment-specific biomarkers, identifying new therapeutic targets, and providing tools to forecast impending MN flares. This would allow for patients to be pre-emptively treated, perhaps without the need for biopsy, shorten exposure to toxic drugs by treating only to the point of immunologic remission and limiting permanent kidney damage.

## Treatment

Treatment of MN has so far focused on non-specific immuno-suppressive therapy, similar to most other autoimmune diseases. Treatment for six months alternating cyclophosphamide with high-dose corticosteroids is still recommended for MN patients that are at very high risk of progression. This ‘gold standard’ in the treatment of MN of cyclic treatment with alkylating agents (cyclophosphamide or chlorambucil) and methyprednisolone, is described in the Ponticelli and ‘modified Ponticelli’ protocols (207) and to date, is the most efficacious at inducing remission. While effective, these cytotoxic drugs have multiple off-target side effects. Rituximab, an anti-CD20 monoclonal antibody for B-cell depletion, has been utilized to induce remission in place of the Ponticelli regimen, with variable efficacy, assessed in various studies comparing with other agents (208–211) and is now recommended for moderate and high risk patients. Calcineurin inhibitors are still a viable alternative to MN patients at moderate risk of progression, but are associated with an increased risk of relapse (212) and dependence.

Corticosteroids, cytotoxic medications, B-cell depletion, and calcineurin inhibitors lack specificity and are potently immunosuppressive. Unraveling the complexity of this disease might lead to more targeted therapy tailored to specific patient needs. Moreover, therapies targeting specific antigens through blockade with mimotopes or interference with synthesis can prove to be beneficial. This may be accomplished through targeting a certain antigen or targeting the epitope *versus* addressing a stressed organelle such as endoplasmic reticulum, or disturbed cytoskeleton, or other methods. Ideally, specific depletion of the responsible clones may result in individualized medicine with improved efficacy and reduced off-target toxicities.

## Discussion

### Future Directions and Unanswered Questions

One challenge to clinically identifying and characterizing MN is the invasive nature of diagnosis by kidney biopsies. The current practice of collecting a renal biopsy is invasive and expensive, and it is impractical for physicians to closely monitor patients using serial biopsies. Future studies will build upon serum and tissue-based proteomic approaches to identify remaining specific biomarkers, potentially enabling development of non-invasive diagnostics and/or prognostic assays. Further characterization of temporal changes in autoantibodies and reactive epitope profiles throughout the different stages of MN is warranted.

Additionally, for some types of MN (EXT1/2 or TGFBR3), it is not yet known whether autoantibodies exist or are these proteins simply biomarkers of disease. The identification of immune complexes eluted from frozen kidney biopsy specimens by tissue IP suggests that a true antigen-antibody interaction exists. Therefore, why are we unable to detect autoantibodies in sera? Antibodies could have very low avidity binding, may circulate in multi-molecular immune complexes where epitopes are opsonized by complement and unavailable for binding, or post-translational modifications *in vivo* are not recapitulated in *in vitro* assays. There may be a mutation resulting in altered protein structure inducing antigenicity, which would not be re-capitulated through use of commercial recombinant proteins. Further work and novel approaches may yield autoantibody detection.

Further understanding of the etiopathogenesis of MN may elucidate further treatment targets. Currently, there is a lack of reliable preclinical models that recapitulate human pathology. To address this issue, future studies will likely introduce mouse models that might allow further investigation of the underlying molecular events of pathological development in this disease.

Ideal therapy in MN should be more specific and tailored to induce tolerance or deplete cells producing the pathogenic antibodies. It is anticipated that future interventional trials will employ two different approaches to target pathological mechanisms in MN. For example, chimeric-antigen-receptor-T-cell (CAR-T) technology will be leveraged to generate specific T cell clones that are able to recognize and destroy B cells expressing antigenic receptors with epitope specificity for target MN antigens (213, 214). Based on proteomic data instigating complement in the pathogenesis of MN (76) and with advances in the therapeutic use of complement inhibitors, it is likely complement inhibition will play a role in the future targeted therapy for MN (215). Peptide-based therapy could also generate mimetics that are able to induce immunological tolerance (216, 217). In this regard, *in vitro* studies of tolerogenic peptides suggested several mechanisms of tolerance induction, such as blocking of MHC class II presentation of the pathogenic peptides by using excess amount of the peptide mimetics or antagonism/partial agonism of TCR signaling and subsequent inhibition of T cell activation (216, 218). This could lead to dramatic improvements in the treatment of MN and reduce drug toxicities.

## Conclusion

In summary, multiple advances have been made in MN over little more than a decade, with the discovery of PLA2R providing the ability to monitor disease. Within only the past three years, a plethora of autoantigens have been identified, reducing our knowledge gap for the protein targets of MN. This could open the door to precision classification through use of algorithmic and multiplex immunostaining, or alternatively, a mass spectrometry-based classification system. Knowledge of autoantigens also opens the door to develop assays for non-invasive monitoring through a serologic-based approach. Additionally, clinical associations of specific antigens with secondary manifestations (malignancy, lupus, and medications) can guide the clinician to evaluate for underlying triggers of the disease. Our understanding of MN pathogenesis is incomplete and further studies will be informative to identify new potential treatment targets through precision medicine. The journey of membranous nephropathy has already reached a new avenue moving away from primary and secondary MN to an antigen-based classification system, enabling diagnosis and monitoring of all types of MN non-invasively, with novel treatments developed from the discoveries.

## Author Contributions

All three authors (LA-R, TC, and LB) contributed equally to the writing of this article, and all authors are accountable for the content of the work.

## Funding

The preparation of this work was supported by institutional funding for the Glomerular Disease Center, Boston Medical Center (LB.) and by the Division of Nephrology & Hypertension, University of Utah Health (LA-R).

## Conflict of Interest

LB reports being a co-inventor on the patent “Diagnostics for Membranous Nephropathy” and receives royalty income through his employer Boston University. LB has served on advisory boards on the topic of MN and other glomerular diseases for Visterra, Ionis, Alexion and Novartis, and receives royalties from UpToDate for topics related to MN.

The remaining authors declare that the research was conducted in the absence of any commercial or financial relationships that could be construed as a potential conflict of interest.

## Publisher’s Note

All claims expressed in this article are solely those of the authors and do not necessarily represent those of their affiliated organizations, or those of the publisher, the editors and the reviewers. Any product that may be evaluated in this article, or claim that may be made by its manufacturer, is not guaranteed or endorsed by the publisher.
